# Kidney histopathology in lethal human sepsis

**DOI:** 10.1186/s13054-018-2287-3

**Published:** 2018-12-27

**Authors:** Adnan Aslan, Marius C. van den Heuvel, Coen A. Stegeman, Eliane R. Popa, Annemarie M. Leliveld, Grietje Molema, Jan G. Zijlstra, Jill Moser, Matijs van Meurs

**Affiliations:** 1 Department of Critical Care, University of Groningen, University Medical Center Groningen, P.O. 30.001, Hanzeplein 1, 9700 RB Groningen, Netherlands; 2 Department of Pathology & Medical Biology, Medical Biology Section, University of Groningen, University Medical Center Groningen, Hanzeplein 1, 9700 RB Groningen, Netherlands; 3 Department of Pathology & Medical Biology, Pathology Section, University of Groningen, University Medical Center Groningen, Hanzeplein 1, 9700 RB Groningen, Netherlands; 4 Department of Nephrology, University of Groningen, University Medical Center Groningen, Hanzeplein 1, 9700 RB Groningen, Netherlands; 5 Department of Urology, University of Groningen, University Medical Center Groningen, Hanzeplein 1, 9700 RB Groningen, Netherlands

**Keywords:** Sepsis, Kidney, AKI, Histopathology, Immune cells, Apoptosis, Fibroblast, Fibrin

## Abstract

**Purpose:**

The histopathology of sepsis-associated acute kidney injury (AKI) in critically ill patients remains an understudied area. Previous studies have identified that acute tubular necrosis (ATN) is not the only driver of sepsis-AKI. The focus of this study was to identify additional candidate processes that may drive sepsis-AKI. To do this we immunohistochemically characterized the histopathological and cellular features in various compartments of human septic kidneys.

**Methods:**

We studied the following histopathological features: leukocyte subsets, fibroblast activation, cellular proliferation, apoptosis, and fibrin deposition in the glomerulus and the tubulointerstitium in human post-mortem kidney biopsy tissue. Biopsy tissue samples from 27 patients with sepsis-AKI were collected 33 min (range 24–150) after death in the ICU. The unaffected part of the kidneys from 12 patients undergoing total nephrectomy as a result of renal carcinoma served as controls.

**Results:**

Immunohistochemical analysis revealed the presence of more neutrophils and macrophages in the glomeruli and more neutrophils in the tubulointerstitium of renal tissue from patients with sepsis compared to control renal tissue. Type II macrophages were predominant, with some macrophages expressing both type I and type II markers. In contrast, there were almost no macrophages found in control kidneys. The number of activated (myo)fibroblasts was low in the glomeruli of sepsis-AKI kidneys, yet this was not observed in the tubulointerstitium. Cell proliferation and fibrin deposition were more pronounced in the glomeruli and tubulointerstitium of sepsis-AKI than in control kidneys.

**Conclusions:**

The extensive heterogeneity of observations among and within patients emphasizes the need to thoroughly characterize patients with sepsis-AKI in a large sample of renal biopsy tissue from patients with sepsis.

**Electronic supplementary material:**

The online version of this article (10.1186/s13054-018-2287-3) contains supplementary material, which is available to authorized users.

## Introduction

Sepsis is a severe and frequent clinical condition in the intensive care unit (ICU) with an associated mortality rate varying between 35 and 50% in septic shock [1–3]. The host response to infection leads to organ failure in patients with sepsis [1]. One of the severely affected organs is the kidney, with sepsis being the leading cause of acute kidney injury (AKI) in critically ill patients [2–4]. Moreover, patients with sepsis-AKI are twice as likely to die as patients with sepsis without AKI [5, 6].

For a long time, acute tubular necrosis (ATN) due to hypoxia together with the severe hyperinflammatory response were thought to be the main drivers of renal failure in patients with sepsis-AKI [4]. Renal tubuli have a marginal oxygen supply combined with high oxygen consumption, yet a reduction in renal blood flow below critical limits is not uniformly reported in clinical sepsis-AKI studies [5]. Additionally, hemodynamic impairment has not been found to be the main driver of renal failure in patients with sepsis [6–8]. During the last three decades numerous clinical trials targeting supposedly pathophysiological mediators within the devastating cascade of inflammatory mediators in sepsis have failed to improve patient outcome. As a result, mortality in sepsis remains unacceptably high [9]. This failure undermines the validity of the hypothetical causality of these mediators in sepsis and underscores the limited understanding of the pathogenesis of sepsis and resulting organ dysfunction [10]. Recently, an elegant sheep model of gram-negative sepsis with intensive care treatment, combined with sequential biopsies, also suggested that hypoxia and inflammation cannot fully explain sepsis-AKI [11]. Histological data addressing morphological damage such as ATN in patients with sepsis-AKI are scarce [12]. In the past, most data were acquired from post-mortem studies conducted hours or even days after death. As a result, tissue autolysis and post-mortem processes may have hampered pathophysiological interpretation [13]. However, recently 2 important studies reported histopathological findings on post-mortem biopsies taken immediately after death [14, 15]. Collectively, the available human tissue data also do not support the hypothesis that hypoxia and inflammation are the major underlying causes of sepsis-AKI, since only limited inflammation, coagulation and cell death have been identified [12, 14, 15]. A better understanding of the underlying pathophysiological mechanisms of AKI in patients with sepsis is indispensable for the development of a therapy that will improve outcome [16, 17]. Therefore, renewed orientation on human tissue with a broader scope might reveal additional pathophysiological mechanisms [13, 18, 19].

The aim of this study was therefore to investigate and expand the histopathological profiles of the glomerular and tubulointerstitial compartments in human sepsis-AKI, including inflammation, coagulation, cell cycle, and repair using immunohistochemical analysis. Compartmentalized detection and quantification of these features may allow us to set the first steps towards therapeutic interventions that more specifically target cell subsets and organ niches in sepsis-AKI.

## Materials and methods

### Patients

We included 27 patients with sepsis-AKI, aged 18 years or older, who had died from sepsis (Table 1 and Additional file 1: Table S1 and S2) between January 2013 and January 2015. All patients were classified as having septic shock and treated accordingly with curative intention [20]. All patients received one or more vasoactive drug. Patients were classified according to the risk, injury, failure, loss, end-stage renal failure (RIFLE) AKI criteria [21]. “Warm” kidney biopsy tissue samples were obtained from these patients within 33 min (range 24–150) after death. Biopsies were performed in 25 patients within 40 min. Messenger RNA (mRNA) levels in a subset of these biopsy samples were previously reported [22, 23]. Exclusion criteria were pre-existing chronic kidney disease (CKD), active autoimmune disorders with renal involvement, and immunosuppressive treatment. CKD was defined in patients on chronic renal replacement therapy or patients with known creatinine clearance below 60 ml/min/1.73 m^2^. Immediate post-mortem biopsies are performed by definition in deceased patients. Therefore, legal regulations for studies in living patients do not apply. We considered our immediate post-mortem biopsies a limited autopsy. Full autopsy was also offered to the relatives of the patients. The limited autopsy was performed by clinicians under the responsibility of the pathologist, with the purpose of exploring the cause of renal failure. Permission and written informed consent for this limited autopsy was asked for in the final family conference before or just after death. The limited autopsy procedure was explained in detail and we explained that we would try to clarify the cause of death and furthermore, that we had a research purpose. An autopsy report of the routine histological findings was added to the patient chart and was discussed during a meeting with the family 6 weeks after ICU admission. Control biopsies were obtained from patients who underwent total nephrectomy as a result of kidney cancer. A healthy section of tissue was isolated from the kidney cortex adjacent to, yet as far away as possible from the tumor. The Medical Ethics Review Committee (METC) of the University Medical Center Groningen (UMCG) reviewed and waived the need for ethics approval for this study (METc 2011/372).

**Table 1:** Patient characteristics

	Sepsis (*n* = 27)	Control (*n* = 12)
Age (mean yearS (range))	67.9 (40–85)	59.5 (20–79)
Sex (female:male)	10:17	7:5
LOS (mean days (range))	3.6 (1–12)	
Comorbidities/Medical history (*n*)		
Hypertension	8	3
Diabetes mellitus	3	1
COPD or asthma	7	4
Coronary disease	5	1
Morbid obesity	2	0
Neurologic	3	1
Renal disease	0	0
Vascular surgery	2	0
Auto-immune disease	4	2
Neoplasms (extra-renal)	4	4
RIFLE stage^a^ (*n*)		
Risk	0	n.a.
Injury	12	
Failure	15	
Lost renal function	0	
End-stage kidney failure	0	
Need for RRT: yes/no	12/15	n.a.
Biopsy time (mean minutes (range))	33 (24–150)	n.a.
Microorganisms (strains)		n.a.
Gram-positive	12	
Gram-negative	23	
Viral (norovirus and HIV)	2	
Fungus/yeast	5	

*LOS* Length of stay, *RRT* renal replacement therapy, *COPD* chronic obstructive pulmonary disease, *HIV* human immunodeficiency virus, *n.a.* not applicable

^a^Number of patients at respective stages

### Harvesting and processing of kidney biopsies

Multiple kidney biopsy tissue samples were harvested from patients with sepsis under ultrasound guidance, after introducing the biopsy device (Angiotech, 14 Ga × 20 cm, Tru Core2 Biopsy Instrument, Gainesville, Fl) through a small (5–7 mm) skin incision. Control renal cortex tissue was obtained by excising the unaffected areas of the kidney that had been removed from patients as a result of renal cell carcinoma. Biopsies were taken within 30 min after kidney removal, which was performed by hand-assisted laparoscopy (*n* = 9) or by the trans-peritoneal (*n* = 2) or trans-lumbar procedure (*n* = 1). Renal biopsy tissues were immediately fixed in 10% formalin fixative for approximately 24 h, which is the recommended fixation time for biopsy tissue without hampering immunohistochemical staining procedures, and were subsequently imbedded in paraffin.

### Histological evaluation of kidney biopsy tissue

For histopathological assessment, deparaffinized sections were stained with hematoxylin and eosin (H&E), periodic acid–Schiff (PAS), and martius, scarlet and blue (MSB). All sections were evaluated by the same experienced nephro-pathologist (MvdH) and counted manually (MvdH and AA). Samples were histopathologically scored manually following the routine pathology procedure in our hospital and therefore could not be blinded. Glomeruli were counted and evaluated for sclerosis, glomerulitis, and an increase in mesangial matrix. Tubuli were evaluated for tubulitis, atrophy, and ATN. The interstitium was evaluated for inflammation and fibrosis. The microvasculature was evaluated for intima sclerosis, intima arteritis, arteriolar hyaline formation and peritubular capillaritis. Fibrin deposition was also determined in kidney biopsy tissue. ATN was scored using the grading system described by Tavares and coworkers [24] and other scoring methods are summarized in Table 2.

**Table 2:** Scoring methods for the histological evaluation of kidney biopsies

Compartment	
Histological feature	
• Scoring method	
Glomerulus	
Total count glomeruli	
• All representative (adequately cross-cut) glomeruli together of 27 patients with sepsis	
Sclerotic glomeruli	
• Scored positive when total sclerosis of glomerulus is observed	
Glomerulitis	
• Yes: ≥ 10 leukocytes in glomerular capillaries	
• No: < 10 leukocytes in glomerular capillaries	
Increase in mesangial matrix	
• Stage 0: no mesangial matrix increase	
• Stage 1: up to 25% of non-sclerotic glomeruli affected (at least moderate matrix increase)	
• Stage 2: 26–50% of non-sclerotic glomeruli affected (at least moderate matrix increase)	
• Stage 3: > 50% of non-sclerotic glomeruli affected (at least moderate matrix increase)	
Leukocyte subsets	
• Number of CD3, CD20, CD68 and neutrophil elastase-positive cells in glomeruli divided by the total count of glomeruli, reported as mean number of positive cells/glomerulus	
Proliferating cells	
• Number of Ki-67 positive cells/glomerulus	
Myofibroblasts	
• Number of anti-α-SMA positive cells in glomeruli divided by the total count of glomeruli, reported as mean positive foci/glomerulus	
Thrombus formation	
• Martius, scarlet and blue positive cells in glomeruli divided by the total count of glomeruli, reported as mean positive foci/glomerulus	
Apoptosis	
• Number of Anti-caspase-3 positive cells in glomeruli divided by the total count of glomeruli, reported as mean positive foci/glomerulus	
Tubulointerstitium	
Tubulitis	
• Stage 0: no mononuclear cells in tubules	
• Stage 1: 1–4 mononuclear cells/tubular cross-section	
• Stage 2: 5–10 mononuclear cells/tubular cross-section	
• Stage 3: > 10 mononuclear cells/tubular cross-section	
Interstitial inflammation	
• Stage 0: no or hardly any interstitial parenchyma covered with mononuclear cells	
• Stage 1: 10–25% of interstitial parenchyma covered with mononuclear cells	
• Stage 2: 26–50% of interstitial parenchyma covered with mononuclear cells	
• Stage 3: > 50% of interstitial parenchyma covered with mononuclear cells	
Interstitial fibrosis	
• Stage 0: interstitial fibrosis tissue of up to 5% of total area	
• Stage 1: mild-interstitial fibrosis tissue of 6–25% of total area	
• Stage 2: moderate-interstitial fibrosis of 26–50% of total area	
• Stage 3: severe interstitial fibrosis of > 50% of total area	
Tubular atrophy	
• Stage 0: no tubular atrophy	
• Stage 1: atrophy in 0–25% of the tubules	
• Stage 2: atrophy in 26–50% of the tubules	
• Stage 3: atrophy of > 50% of the tubules	
Leukocyte subsets	
Number of CD3, CD20, CD68 and neutrophil elastase-positive cells	
• Stage 0: absent	
• Stage 1: focally present	
• Stage 2: diffusely present	
Proliferating cells	
• Number of Ki-67 positive cells/200 tubular epithelial cells	
Myofibroblasts	
anti-α-SMA positive cells present in tubulointerstitium	
• Stage 0: none	
• Stage 1: present in 0–25% of tubulointerstitium	
• Stage 2: present in 26–50% of tubulointerstitium	
• Stage 3: present in 51–75% of tubulointerstitium	
• Stage 4: present in > 75% of tubulointerstitium	
Thrombus formation	
• Number of martius, scarlet and blue total positive foci/2 mm^2^	
Apoptosis	
Number of Anti-caspase-3 total positive cells present in tubulointerstitium	
• Stage 0: absent	
• Stage 1: present in 0–10% of total interstitial cells	
• Stage 2: present in 11–50% of total interstitial cells	
• Stage 3: present in > 50% of total interstitial cells	
Blood vessels	
Arteriolar intima sclerosis	
• Stage 0: no chronic vascular changes	
• Stage 1: vascular narrowing of up to 25% luminal area of arteries fibro-intimal thickening	
• Stage 2: vascular narrowing with 26–50% luminal area of arteries fibro-intimal thickening	
• Stage 3: vascular narrowing with 26–50% luminal area of arteries fibro-intimal thickening	
Arteriolar hyaline	
• Stage 0: no PAS-positive hyaline thickening	
• Stage 1: mild-to-moderate PAS-positive hyaline in at least one arteriole	
• Stage 2: moderate-to-severe PAS-positive hyaline thickening in more than one arteriole	
• Stage 3: severe PAS-positive hyaline thickening in many arterioles	
Intima arteritis	
• Stage 0: no arteritis	
• Stage 1: mild-to-moderate intimal arteritis in at least one arterial cross-section	
• Stage 2: severe intima arteritis with at least 25% luminal area lost in at least one arterial cross-section	
• Stage 3: arterial fibrinoid change and/or transmural arteritis with medial smooth muscle necrosis with lymphocytic inflammation	
Peritubular capillaritis	
• Stage 0: < 10% of capillaries contain inflammatory cells	
• Stage 1: > 10% of capillaries contain inflammatory cells, 3–4 mononuclear cells in peritubular capillary lumen	
• Stage 2: > 10% of capillaries contain inflammatory cells, 5–10 mononuclear cells in peritubular capillary lumen	
• Stage 3: if > 10% of capillaries contain inflammatory cells, > 10 mononuclear cells in peritubular capillary lumen	

*PAS* periodic acid–Schiff, *SMA* smooth muscle actin

### Immunohistochemical analysis

For immunohistochemical analysis, tissue sections (4 μm) were deparaffinized and rehydrated, and endogenous peroxidase activity was blocked following standard protocols. Samples were immunohistochemically stained to detect neutrophils (neutrophil elastase), macrophages (CD68), T lymphocytes (CD3, CD4, CD8), B lymphocytes (CD20), myofibroblasts (alpha-smooth muscle actin (SMA)), proliferating cells (Ki-67), and apoptotic cells (activated caspase 3). Staining was performed in a Benchmark Ultra automated IHC/ISH slide staining system (Ventana Medical Systems, Roche Diagnostics, Almere, The Netherlands). This system is used for diagnostic purposes in our pathology department. The staining system and the ready-to-use antibodies were validated by the manufacturer and verified in our pathology department. Control tissue (appendix tissue) was routinely added for each staining procedure. Briefly, antigens were retrieved by boiling the sections in 10 mM Tris/1 mM EDTA pH 9.0 for 15 min. Sections were incubated with primary antibodies (Additional file 2: Table S3) for 1 h, and subsequently with properly matching secondary HRP-conjugated antibodies (DAKO, Carpentaria, CA, USA) for 45 min. Standard washing steps were performed between incubations. Peroxidase activity was detected using 3-amino-9-ethylcarbazole (DAKO).

To detect type I and type II macrophages, antigens were retrieved by boiling the sections for 15 min in 0.1 M (type I macrophages) or 10 mM (type II macrophages) Tris/1 mM EDTA pH 9.0. Endogenous biotin was blocked using the avidin/biotin blocking kit (Vector Laboratories, Burlingame, CA, USA). Sections were sequentially incubated for 1 h at room temperature (RT) with primary antibodies against human CD68 and IRF5 to identify type I macrophages, or with primary antibodies against human CD68 and CD163 to identify type II macrophages [25]. Sections were subsequently incubated for 1 h at RT with goat anti-rabbit-alkaline phosphatase (DAKO) and goat anti-mouse-HRP (DAKO) for type I macrophages, or goat anti-mouse IgG3-Biotin and goat anti-mouse IgG1-HRP (Southern Biotech, Birmingham, AL 35260, USA) for type II macrophages. Sections stained for type II macrophages were incubated with streptavidin-alkaline phosphatase (DAKO) after washing. Alkaline phosphatase and peroxidase activity were detected using the BCIP/NBT kit (Thermo Fisher Scientific, Waltham, MA, USA) and the NovaRed kit (Vector Laboratories), respectively. Sections were counterstained with Mayer’s hematoxylin (Merck, Darmstadt, Germany), mounted and scanned using a Hamamatsu Nanozoomer 2.0 HT (Hamamatsu Photonics, Hamamatsu, Japan). Representative images were captured at x 40 magnification.

### Statistical analysis

Data were analyzed by the two-tailed Mann–Whitney test using Graphpad Prism software v7 (GraphPad Prism software Inc., San Diego, CA, USA) . Differences were considered significant when the *p* value was < 0.05.

## Results

### Histopathological characterization

We first evaluated 926 glomeruli from the kidney biopsy tissues for signs of glomerulosclerosis, glomerulitis, and mesangial matrix expansion. All glomeruli were considered representative. The number of glomeruli differed per individual biopsy (range 11–85) but was not significantly different between patients with sepsis and control subjects (data not shown). Only a few glomeruli (31/926) in renal biopsies from patients with sepsis had sclerotic changes (Additional file 2: Table S4). Glomerulitis was absent and an increase in mesangial matrix was sporadically noted in the glomeruli in samples from patients with sepsis-AKI.

Tubulitis, defined as the presence of inflammatory cells in the tubular wall, was absent. However, interstitial inflammation was observed in two patients, fibrosis in seven, and tubular atrophy in eight patients with sepsis-AKI (Additional file 2: Table S4). Leukocytes were predominantly located within the peritubular capillaries. ATN was discontinuously distributed in parts of the renal biopsy from 24/27 patients with sepsis and when present was in the range of 5–20% of the total tissue area (Additional file 2: Table S5). Most of these patients had morphology stage 2 ATN, where vacuolization, tubular edema, epithelial flattening, and some apoptotic tubular cells were present. Twelve patients with sepsis received renal replacement therapy (RRT) during their stay in the ICU.

Vascular intima sclerosis was observed in 20 sepsis biopsy samples (stage 1, *n* = 13; stage 2, *n* = 7; Additional file 2: Figure S1). Arteriolar hyaline formation was observed in 14 patients (stage 1, *n* = 12; stage 2, *n* = 2). Intima arteritis was absent. Histopathological changes were absent in control biopsy tissues.

### Infiltration of inflammatory cells

**Fig. 1:**
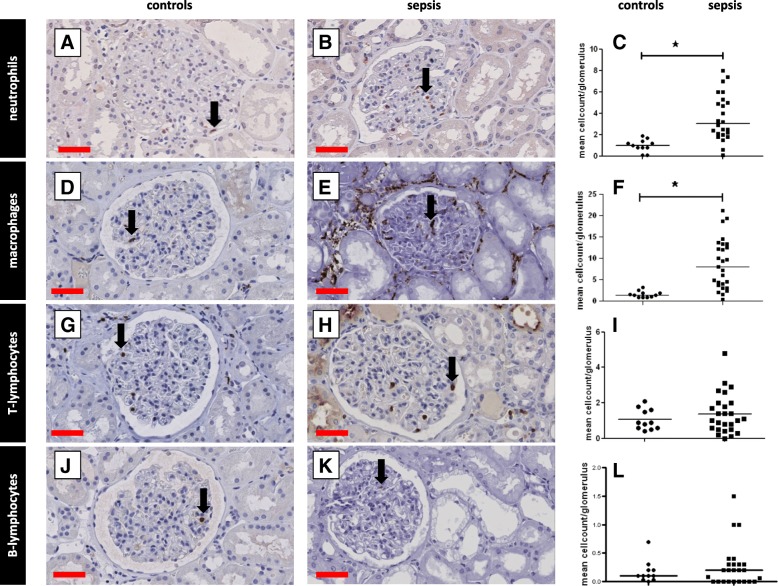
Leukocyte infiltration in the glomeruli. Leukocyte subsets were immunohistochemically detected in kidney biopsy samples using specific antibodies (Additional file 2: Table S3: Primary antibodies) and scored according to Table 2. Leukocyte subsets were counted and divided by the number of glomeruli per patient. Neutrophils (**a**, **b**), macrophages (**d**, **e**), T lymphocytes (**g**, **h**), and B lymphocytes (**j**, **k**) were observed in the glomeruli in kidney tissue from control patients (**a**, **d**, **g**, **j**) and patients with sepsis (**b**, **e**, **h**, **k**), and mean leukocyte counts were determined (**c**, **f**, **i**, **l**). Black arrows show positively stained leukocytes of various subsets. *Statistically significant. Black lines are medians (**c**, **f**, **i, l**). Red scale bar = 50 μm

Since inflammation is a classic hallmark of sepsis, we investigated whether there was inflammatory cell infiltration within the glomerular and tubulointerstitial compartments of the kidney in sepsis-AKI. There were significantly more neutrophils (*p* < 0.0001; Fig. 1 A-C) and macrophages (*p* < 0.0001; Fig. 1 D-F) within the glomeruli in sepsis-AKI compared to controls. Glomerular infiltration of CD3-positive T lymphocytes (Fig. 1 G-I) and B-lymphocytes (Fig. 1 J-L) was low and did not differ between sepsis-AKI and control samples. As a consequence, the CD4/CD8 ratio within the glomeruli could not be determined.

**Fig. 2:**
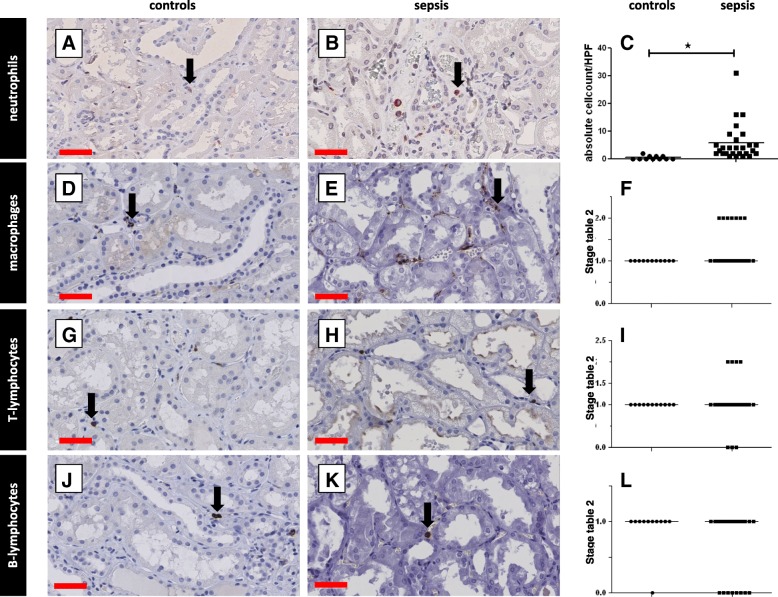
Leukocyte infiltration in the tubulointerstitium. Leukocyte subsets were immunohistochemically detected in kidney biopsy samples using specific antibodies (Additional file 2: Table S3: Primary antibodies) and scored according to Table 2. Neutrophils (**a**, **b**), macrophages (**d**, **e**), T lymphocytes (**g**, **h**), and B lymphocytes (**j**, **k**) were observed in the interstitium in kidney samples from control patients (**a**, **d**, **g**, **j**) and patients with sepsis (**b**, **e**, **h**, **k**), and mean leukocyte counts were determined (**c**, **f**, **i**, **l**). T lymphocytes, B lymphocytes, and macrophages were quantified using an arbitrary score (0 = absent, 1 = focal, 2 = diffuse; **c**, **f**, **i**). For neutrophils, absolute cell counts were determined per high power field (HPF). Black arrows show stained leukocytes of various subsets. *Statistically significant difference. Black lines are medians (**c**, **f**, **i**, **l**). Red scale bars = 50 μm

There was significantly more neutrophil infiltration in the tubulointerstitium in sepsis-AKI (*p* < 0.0001; Fig. 2 A-C), paralleling the glomerular findings. However, in contrast to observations in the glomerulus, macrophage infiltration was similar in both sepsis-AKI and controls (*p* = 0.19; Fig. 2 D-F). Moreover, there was no difference in T lymphocyte (Fig. 2 G-I) and B lymphocyte counts (Fig. 2 J-L) in sepsis-AKI and controls. There were more neutrophils in the peritubular capillaries in sepsis-AKI compared to controls, but no difference was found in lymphocyte (T and B) or macrophage counts (Fig. 2 D-L).

### Intrarenal presence of repair cells

**Fig. 3:**
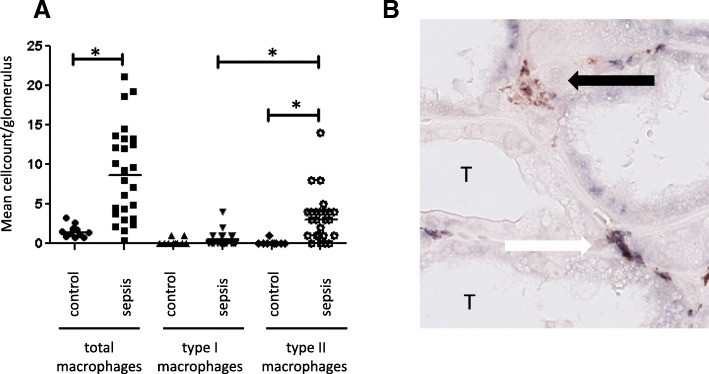
Macrophage subtypes in sepsis with acute kidney injury. Using specific antibodies (Additional file 2: Table S3: Primary antibodies) and scored according to Table 2A,: total macrophages (CD68) and macrophage subtypes (type I: IRF-5, type II: CD163) were immunohistochemically detected and quantified in the glomeruli in kidney samples from patients with sepsis and controls. **a** Representative picture of a tubular region of a biopsy from a patient with sepsis to illustrate the morphology of an intermediate macrophage. Black arrow: type II macrophage in the peritubular capillary (CD163-positive). White arrow: “intermediate” macrophage with double staining positive for IRF-5 (type I macrophage) and CD163 (type II macrophage) in the peritubular capillary (**b**). *Statistically significant differences. Black lines are medians T, tubulus

Since we found increased numbers of macrophages in the glomeruli in sepsis-AKI compared to control biopsy tissues, we proceeded to investigate whether these cells participate in tissue destruction or renal repair. Macrophages can be classified into subtypes, with type I being involved in inflammation and tissue destruction, and type II in tissue repair [26–29]. Macrophage subtype analysis in the glomeruli in samples indicated that patients with sepsis predominantly had type II macrophages (Fig. 3A). Some macrophages in the glomeruli and tubulointerstitium from 12 patients with sepsis-AKI expressed both type I and type II markers (Fig. 3B).

**Fig. 4:**
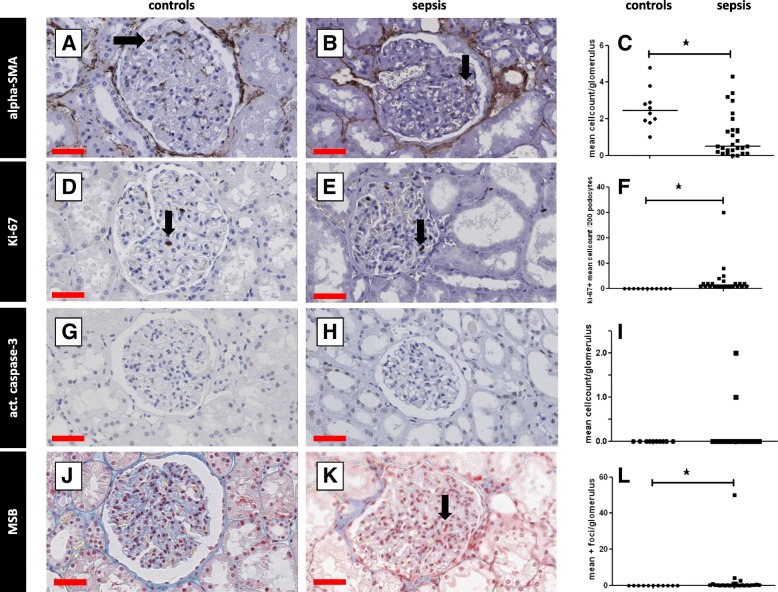
Proliferation, fibrin deposition and apoptosis in the glomeruli. Markers for tissue repair and fibrin deposition were immunohistochemically detected in kidney biopsy samples using specific antibodies (Additional file 2: Table S3: Primary antibodies) and scored according to Table 2. Myofibroblasts (alpha-smooth muscle actin (SMA; **a**, **b**), cell proliferation (Ki-67; **d**, **e**), apoptosis (caspase 3; **g**, **h**), and fibrin deposition (martius, scarlet, and blue; **j**, **k**) were detected in the glomeruli in samples from control patients (**a**, **d**, **g**, **j**) and patients with sepsis (**b**, **e**, **h**, **k**) and quantified (**c**, **f**, **i**, **l**) per podocyte per patient or per glomerulus per patient. Black arrows show representative staining. *Statistically significant difference. Black lines are medians (**c**, **f**, **i**, **l**). Red scale bar = 50 μm

**Fig. 5:**
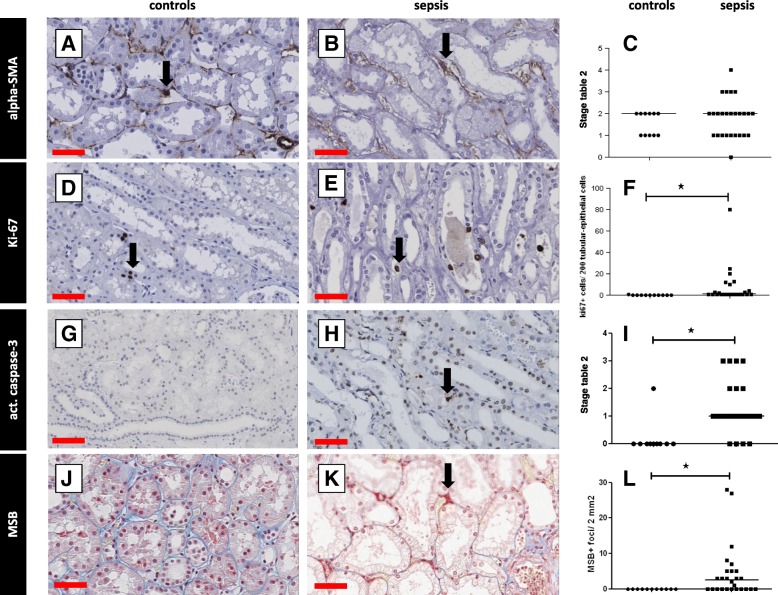
Proliferation, fibrin deposition and apoptosis in the tubulointerstitium. Markers for tissue repair and fibrin deposition were immunohistochemically detected in kidney biopsy samples using specific antibodies (Additional file 2: Table S3: Primary antibodies) and scored according to Table 2. Myofibroblasts (alpha-smooth muscle actin (SMA); **a**, **b**), cell proliferation (Ki-67; **d**, **e**), apoptosis (caspase 3; **g**, **h**), and fibrin deposition (martius, scarlet, and blue; **j**, **k**) were detected in the interstitium in samples from control patients (**a**, **d**, **g**, **j**) and patients with sepsis (**b**, **e**, **h**, **k**) and quantified (**c**, **f**, **i**, **l**). Caspase staining was quantified using an arbitrary score (0, absent; 1, > 0–10% cells positive; 2, > 10–50% cells positive; 3, > 50% cells positive). Black arrows show representative staining. *Statistically significant difference. Black lines are medians (**c**, **f**, **i**, **l**). Red scale bar = 50 μm

We additionally investigated whether α-SMA-expressing myofibroblasts, involved in scar formation, were present. There were significantly fewer myofibroblasts in the glomeruli in samples from patients with sepsis-AKI compared to controls (*p* < 0.0025; Fig. 4 A-C). There was no difference in the number of α-SMA-positive cells in the tubulointerstitium in sepsis-AKI and control kidneys (Fig. 5A, B, and C).

### Proliferation and apoptosis in renal sepsis

Pursuing further the question of renal damage and repair, we investigated whether cell proliferation and apoptosis would be prominent in the kidney in patients with sepsis-AKI. There were significantly more proliferating cells in both the glomeruli (*p* < 0.0001; Fig. 4 D-F), and the tubulointerstitium (*p* < 0.0001; Fig. 5 D-F) in sepsis-AKI than in controls.

Apoptotic cells were virtually absent in the glomeruli in sepsis-AKI (in total 3 apoptotic cells/26 patients) and controls (Fig. 4 G-I). In contrast, there were significantly more tubulointerstitial apoptotic cells in sepsis-AKI compared to control renal tissue (*p* < 0.001; Fig. 5 G-I). However, apoptotic cells were absent in the tubulointerstitium in samples from four patients with sepsis-AKI. We only found apoptotic cells (10–50%) in one control patient.

### Fibrin deposition

The formation of microvascular fibrin deposition due to reduced blood flow and/or endothelial activation might be another deleterious event in sepsis-AKI. Fibrin-stained thrombi were identififed in the glomeruli in sepsis-AKI (*p* < 0.0002), but not in controls (Fig. 4 J, K). One patient with sepsis had signs of disseminated intravascular coagulation with abundant fibrin deposition.

Fibrin-stained thrombi in the tubulointerstitium were predominantly present in the peritubular capillary network in 16/27 patients with sepsis-AKI (*p* < 0.0002; Fig. 5 J-L). Some patients had abundant capillary fibrin deposition. Fibrin deposition was absent in the peritubular capillaries in control renal tissue.

## Discussion

The failure of clinical trials that aim to improvwe the outcome of patients with sepsis-AKI stresses the need for a more detailed understanding of the pathophysiological processes underlying the development of human sepsis-AKI. The aim of this study was therefore to investigate the missing histopathological information on the glomerular and tubulointerstitial compartments in sepsis-AKI, such as renal inflammation, fibrin deposition, cell proliferation, and repair.

The results from our study corroborate findings from recent animal and human studies that show that sepsis-AKI cannot be explained solely by morphological changes. The landmark study by Takasu and colleagues showed that tubular injury in sepsis was common but focal [14]. Moreover, in a sheep model in which sepsis was induced by continuous intravenous infusion of live bacteria, the anatomical structure of the kidney was intact [30]. We found renal tubular damage was unequally distributed and limited in sepsis-AKI. These findings agree with the Takasu study and support the premise that tubular damage cannot fully explain the renal function impairment often found in patients with sepsis [14]. Moreover, our findings also support those found by Lerolle and colleagues in which they demonstrated that AKI in 19 patients with sepsis was associated with intense infiltration of glomeruli, interstitial capillaries, and occasionally tubular lumens, by predominantly monocytic leucocytes [15].

Interestingly, the glomerular architecture was hardly affected in sepsis-AKI. While structural defects were not apparent, the molecular mechanisms regulating endothelial permeability, one of the determining factors of glomerular filtration, may explain the decreased glomerular filtration rate in these patients. Our previous studies identified altered mRNA levels of the endothelial molecules regulating vascular permeability in this cohort of patients [22, 23], and in animal models of lipopolysaccharide (LPS)-induced AKI [23].

Although apoptosis is considered one of the main mechanisms of tubular damage in AKI, only 21 of the 102 experimental studies on sepsis-AKI focused on apoptosis [31]. In these studies, 158/170 animals had tubular epithelial cell apoptosis. In previous studies of patients with sepsis there were only slight increases in the amount of apoptotic tubulointerstitial cells [12, 13], or the absence thereof [32], suggesting a limited role for apoptosis in sepsis. Lerolle and colleagues observed apoptosis of tubular cells and occasionally glomerular cells in patients with sepsis [15]. In contrast, in sepsis-AKI we identified a varying (up to high) extent of apoptosis in the tubulointerstitium but not in the glomeruli. Our findings, and the discrepancy between animal and human studies, suggest that the role of apoptosis in the tubulointerstitial compartment needs to be revisited by expanding these types of studies in human AKI before rightful conclusions on its role in AKI can be reached.

Local microvascular thrombosis, a possible consequence of low flow and/or microvascular endothelial activation [33], might be another mechanism propagating tissue damage in sepsis-AKI. We found low amounts of fibrin deposition in the glomeruli, similar to Lerolle et al., who observed glomerular fibrin depositions in only 1/19 patients with sepsis [15]. In contrast, while we found abundant fibrin deposition in the peritubular capillaries, these authors report partial or complete thrombi in the afferent arterioles in four patients, but no significant fibrin deposition in the peritubular capillary system [13]. Similarly, Takasu et al. did not find fibrin deposition in peritubular capillaries in post-mortem kidneys from patients with sepsis [14].

Among the cellular players held responsible for tissue damage in sepsis-AKI, neutrophils are the first to infiltrate the tissues. Neutrophil blockade or depletion in experimental animal models have varying results in preventing AKI [34–36]. Here, we found a notable but limited number of neutrophils in the glomeruli and tubulointerstitium, including in the peritubular capillaries. However, no renal cell injury could be attributed to this neutrophil influx. Lerolle et al. reported that neutrophil infiltration was limited to the peritubular capillaries [15]. Neutrophil function is ambiguous as these cells are also required for tissue regeneration [34, 37–39]. Which neutrophil function prevails in human sepsis-AKI is an important future research focus.

Lymphocytes play an important role in the cytokine storm characterizing sepsis. In murine endotoxemia, T lymphocytes were shown to be modulators of kidney function and responsible for renal neutrophil recruitment [40]. Moreover, T-lymphocytes are important in the development of AKI in experimental ischemia reperfusion injury (IRI) [41]. T lymphocytes were not found to be important in sepsis-AKI in our study, as these cells hardly invaded the kidney. The role of B lymphocytes in sepsis-AKI has not been studied extensively. The limited experimental evidence available suggests that B lymphocytes may slow down or limit the repair process of the kidney after an ischemic insult. Moreover, B-lymphocyte deficiency was shown to be protective against ischemia reperfusion injury [42, 43]. We hardly found any B lymphocyte infiltration in the kidneys of patients with sepsis-AKI. Together these findings suggest that inflammatory damage mediated by B lymphocytes or T lymphocytes seems to play a minor role in human sepsis-AKI. Nevertheless, functional contributions of these cells cannot be excluded in this observational study.

The dual role of macrophages in damage and repair in sepsis-AKI is elusive. Renal mononuclear phagocytic cells consist of different subtypes, of which type I macrophages are involved in inflammation and tissue destruction, while type II macrophages attenuate the inflammatory response and are active during tissue repair [26, 27, 29]. We observed an accumulation of macrophages of both subtypes around the glomeruli and in the glomerular capillaries, but not in the tubulointerstitium of patients with sepsis-AKI. The immediate vicinity of both macrophage subtypes may suggest inflammation and repair being simultaneously active in time and space. Type II macrophages in sepsis-AKI glomeruli may originate from the systemic circulation, but also from activated, proliferating local cells [44]. We also found occasional macrophages expressing markers of both macrophage subtypes. A similar observation was previously described in the kidneys of rats with glycol-induced rhabdomyolysis [45]. These cells were proposed to transition from type I to type II and were shown to increase in numbers during recovery [45].

We also investigated cell proliferation as a marker of tissue repair. We found increased proliferation in glomeruli and tubulointerstitium of sepsis kidneys, concurring with the findings of Takasu et al. [14]. Although the identity of proliferating cells was not explored, their presence suggests increased regeneration of renal tissue as a mechanism of repair. Interestingly, the amount of α-SMA-expressing myofibroblasts, which initially contribute to repair through matrix deposition, was reduced in sepsis-AKI glomeruli and unchanged in the tubulointerstitium. Sandbo et al., identified inhibition of α-SMA expression at the protein and mRNA level in LPS-stimulated vascular smooth muscle cells of the human aorta, human coronary artery, and rat aorta [46]. They found that decreased levels of α-SMA correlate with diminished cellular contractile function. Renal scar tissue formation and fibrosis was not abundant in sepsis-AKI although obviously, long-term effects were not studied. The role of repair mechanisms in sepsis-AKI is understudied but preliminary data showing signs of overactivity and underactivity justify further research.

The strength of this current study lies in the early retrieval of kidney biopsy tissues directly post mortem, minimizing the influence of autolysis on subsequent analyses. Indeed, our current and previous studies and studies by others [22, 23, 47, 48] show the feasibility of advanced immunohistochemical, mRNA, proteomic, and metabolomic analyses of these biopsies. When integrated, the resulting datasets will allow better and extensive patient characterization and will hopefully pave the way towards precision and even personalized medicine in sepsis-AKI [19].

Despite this future promise, our study has limitations, mainly the heterogeneity of patients with sepsis-AKI and therefore the number of included patients. To correlate our heterogeneous findings with the heterogeneous clinical characteristics of our patients and draw meaningful conclusions is not possible. Large data and tissue banks will be required for multivariate analyses. Most patients with sepsis are relatively old and vary widely in clinical presentation. Comorbidities and causative microorganisms influence renal pathophysiology. Additionally, some patients may have had unknown previous renal disease. We must emphasize that the time between the start of sepsis and death varied considerably between patients. Additionally, the biopsies were taken after a prolonged, variable process of illness and dying, both of which might have interfered with the observed AKI-related changes. We only studied biopsy tissues from deceased patients, which confines our results to the sickest patient group. Control patients have comorbidity as well, and the surgical procedure to procure the kidney with clamping of the renal artery might have already induced histopathological changes [49]. The choice of control group can be debated. Furthermore, it is of note that all biopsies were reviewed by one experienced renal pathologist which improves consistency but interobserver variability is well-known among pathologists. Moreover, the histopathologic scoring followed the routine pathology procedure in our hospital and therefore could not be blinded, which may have introduced some observer bias. We also do not know whether our pathophysiological findings in septic kidneys are the effect of renal processes or processes elsewhere in the body. Our observations described here might be an epiphenomenon, which makes the question “What causes renal function loss in sepsis?” even more enigmatic.

## Conclusion

Our study investigating the histopathology of sepsis-AKI revealed heterogenic pathophysiological processes among patients. No one pathogenic mechanism can be singled out. Our results imply a spectrum of overactive and underactive biological processes.

## Additional files

Additional file 1: Patient and control characteristics. (PPTX 96 kb)

Additional file 2: Supplementary methods and results. (PDF 78 kb)

## Abbreviations

AKI: Acute kidney injury
ATN: Acute tubulus necrosis
H&E: Hematoxylin and eosin
ICU: Intensive care unit
IRI: Ischemia reperfusion injury
LPS: Lipopolysaccharide
MSB: Martius, scarlet, and blue
PAS: Periodic acid–Schiff
RIFLE: Risk, injury, failure, loss, end-stage renal failure
RRT: Renal replacement therapy
