# Supplementary material for: Kidney histopathology in lethal human sepsis
Source: Crit Care. 2018 Dec 27;22:359. doi: 10.1186/s13054-018-2287-3 (PMC6307291; doi:10.1186/s13054-018-2287-3)
Supplement: Supplementary file 1 — Patient and control characteristics. (PPTX 96 kb) [file 13054_2018_2287_MOESM1_ESM.pptx]

## Slide 1
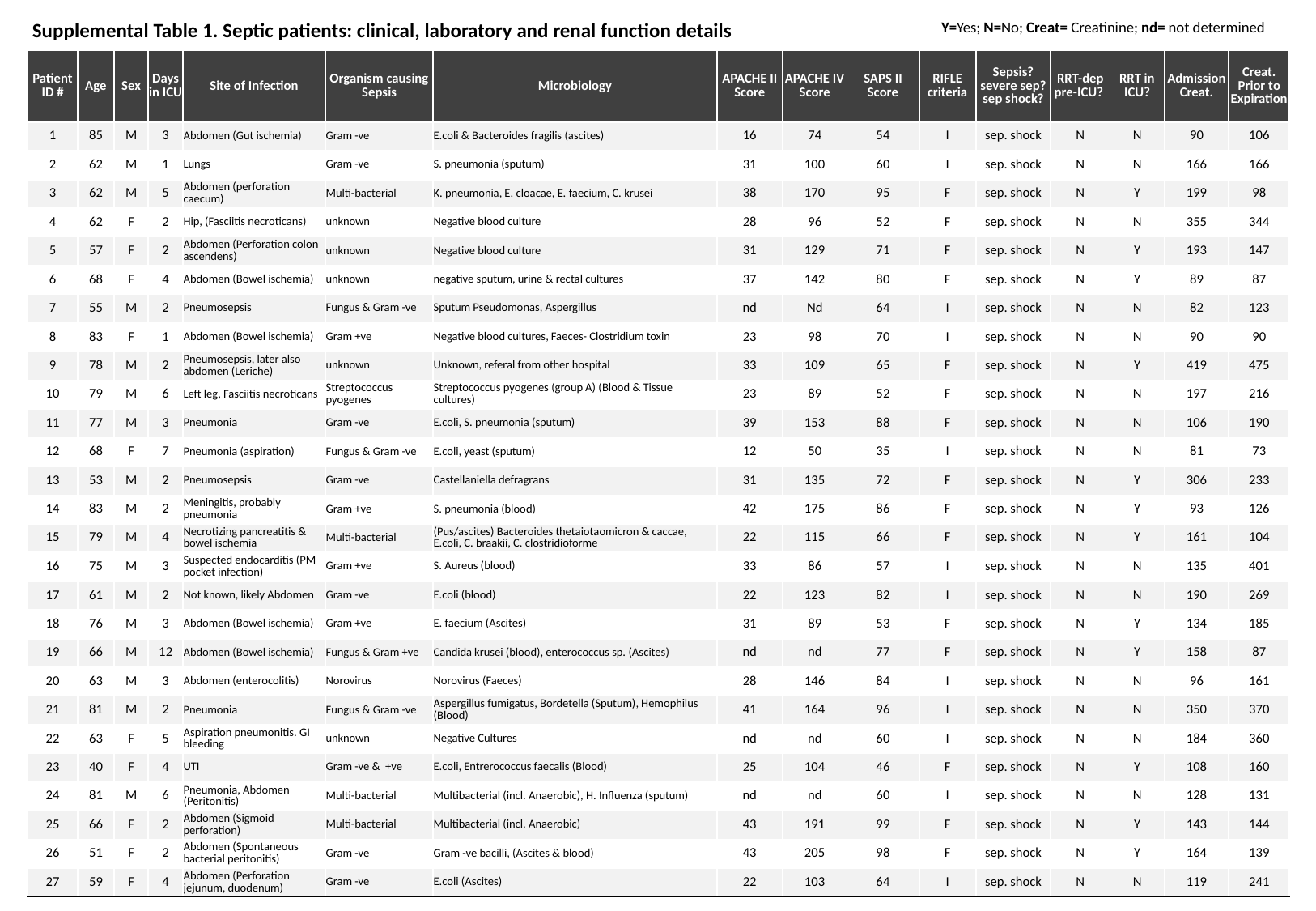

Y=Yes; N=No; Creat= Creatinine; nd= not determined
Supplemental Table 1. Septic patients: clinical, laboratory and renal function details
| Patient ID # | Age | Sex | Days in ICU | Site of Infection | Organism causing Sepsis | Microbiology | APACHE II Score | APACHE IV Score | SAPS II Score | RIFLE criteria | Sepsis? severe sep? sep shock? | RRT-dep pre-ICU? | RRT in ICU? | Admission Creat. | Creat. Prior to Expiration |
| --- | --- | --- | --- | --- | --- | --- | --- | --- | --- | --- | --- | --- | --- | --- | --- |
| 1 | 85 | M | 3 | Abdomen (Gut ischemia) | Gram -ve | E.coli & Bacteroides fragilis (ascites) | 16 | 74 | 54 | I | sep. shock | N | N | 90 | 106 |
| 2 | 62 | M | 1 | Lungs | Gram -ve | S. pneumonia (sputum) | 31 | 100 | 60 | I | sep. shock | N | N | 166 | 166 |
| 3 | 62 | M | 5 | Abdomen (perforation caecum) | Multi-bacterial | K. pneumonia, E. cloacae, E. faecium, C. krusei | 38 | 170 | 95 | F | sep. shock | N | Y | 199 | 98 |
| 4 | 62 | F | 2 | Hip, (Fasciitis necroticans) | unknown | Negative blood culture | 28 | 96 | 52 | F | sep. shock | N | N | 355 | 344 |
| 5 | 57 | F | 2 | Abdomen (Perforation colon ascendens) | unknown | Negative blood culture | 31 | 129 | 71 | F | sep. shock | N | Y | 193 | 147 |
| 6 | 68 | F | 4 | Abdomen (Bowel ischemia) | unknown | negative sputum, urine & rectal cultures | 37 | 142 | 80 | F | sep. shock | N | Y | 89 | 87 |
| 7 | 55 | M | 2 | Pneumosepsis | Fungus & Gram -ve | Sputum Pseudomonas, Aspergillus | nd | Nd | 64 | I | sep. shock | N | N | 82 | 123 |
| 8 | 83 | F | 1 | Abdomen (Bowel ischemia) | Gram +ve | Negative blood cultures, Faeces- Clostridium toxin | 23 | 98 | 70 | I | sep. shock | N | N | 90 | 90 |
| 9 | 78 | M | 2 | Pneumosepsis, later also abdomen (Leriche) | unknown | Unknown, referal from other hospital | 33 | 109 | 65 | F | sep. shock | N | Y | 419 | 475 |
| 10 | 79 | M | 6 | Left leg, Fasciitis necroticans | Streptococcus pyogenes | Streptococcus pyogenes (group A) (Blood & Tissue cultures) | 23 | 89 | 52 | F | sep. shock | N | N | 197 | 216 |
| 11 | 77 | M | 3 | Pneumonia | Gram -ve | E.coli, S. pneumonia (sputum) | 39 | 153 | 88 | F | sep. shock | N | N | 106 | 190 |
| 12 | 68 | F | 7 | Pneumonia (aspiration) | Fungus & Gram -ve | E.coli, yeast (sputum) | 12 | 50 | 35 | I | sep. shock | N | N | 81 | 73 |
| 13 | 53 | M | 2 | Pneumosepsis | Gram -ve | Castellaniella defragrans | 31 | 135 | 72 | F | sep. shock | N | Y | 306 | 233 |
| 14 | 83 | M | 2 | Meningitis, probably pneumonia | Gram +ve | S. pneumonia (blood) | 42 | 175 | 86 | F | sep. shock | N | Y | 93 | 126 |
| 15 | 79 | M | 4 | Necrotizing pancreatitis & bowel ischemia | Multi-bacterial | (Pus/ascites) Bacteroides thetaiotaomicron & caccae, E.coli, C. braakii, C. clostridioforme | 22 | 115 | 66 | F | sep. shock | N | Y | 161 | 104 |
| 16 | 75 | M | 3 | Suspected endocarditis (PM pocket infection) | Gram +ve | S. Aureus (blood) | 33 | 86 | 57 | I | sep. shock | N | N | 135 | 401 |
| 17 | 61 | M | 2 | Not known, likely Abdomen | Gram -ve | E.coli (blood) | 22 | 123 | 82 | I | sep. shock | N | N | 190 | 269 |
| 18 | 76 | M | 3 | Abdomen (Bowel ischemia) | Gram +ve | E. faecium (Ascites) | 31 | 89 | 53 | F | sep. shock | N | Y | 134 | 185 |
| 19 | 66 | M | 12 | Abdomen (Bowel ischemia) | Fungus & Gram +ve | Candida krusei (blood), enterococcus sp. (Ascites) | nd | nd | 77 | F | sep. shock | N | Y | 158 | 87 |
| 20 | 63 | M | 3 | Abdomen (enterocolitis) | Norovirus | Norovirus (Faeces) | 28 | 146 | 84 | I | sep. shock | N | N | 96 | 161 |
| 21 | 81 | M | 2 | Pneumonia | Fungus & Gram -ve | Aspergillus fumigatus, Bordetella (Sputum), Hemophilus (Blood) | 41 | 164 | 96 | I | sep. shock | N | N | 350 | 370 |
| 22 | 63 | F | 5 | Aspiration pneumonitis. GI bleeding | unknown | Negative Cultures | nd | nd | 60 | I | sep. shock | N | N | 184 | 360 |
| 23 | 40 | F | 4 | UTI | Gram -ve & +ve | E.coli, Entrerococcus faecalis (Blood) | 25 | 104 | 46 | F | sep. shock | N | Y | 108 | 160 |
| 24 | 81 | M | 6 | Pneumonia, Abdomen (Peritonitis) | Multi-bacterial | Multibacterial (incl. Anaerobic), H. Influenza (sputum) | nd | nd | 60 | I | sep. shock | N | N | 128 | 131 |
| 25 | 66 | F | 2 | Abdomen (Sigmoid perforation) | Multi-bacterial | Multibacterial (incl. Anaerobic) | 43 | 191 | 99 | F | sep. shock | N | Y | 143 | 144 |
| 26 | 51 | F | 2 | Abdomen (Spontaneous bacterial peritonitis) | Gram -ve | Gram -ve bacilli, (Ascites & blood) | 43 | 205 | 98 | F | sep. shock | N | Y | 164 | 139 |
| 27 | 59 | F | 4 | Abdomen (Perforation jejunum, duodenum) | Gram -ve | E.coli (Ascites) | 22 | 103 | 64 | I | sep. shock | N | N | 119 | 241 |

## Slide 2
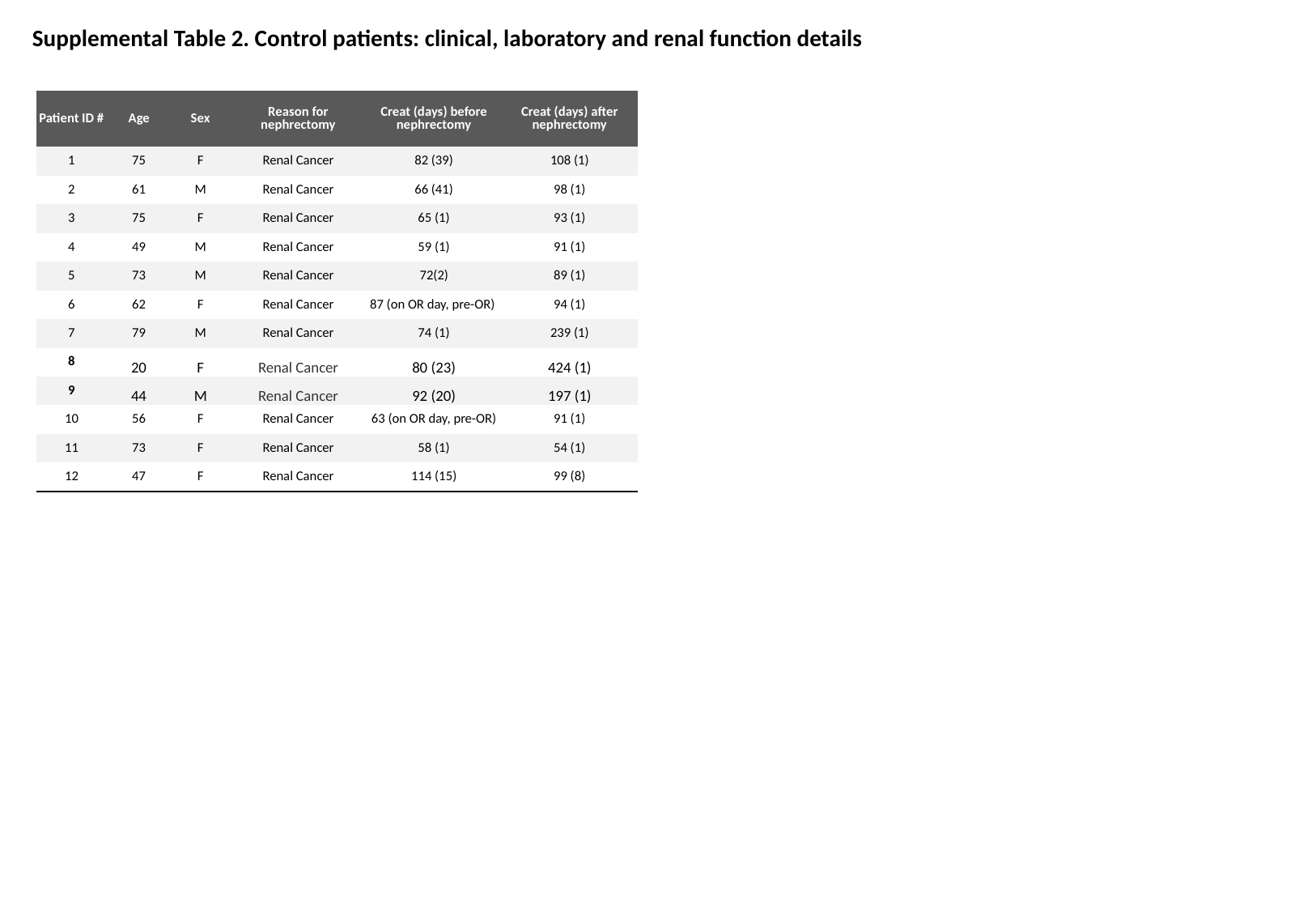

Supplemental Table 2. Control patients: clinical, laboratory and renal function details
| Patient ID # | Age | Sex | Reason for nephrectomy | Creat (days) before nephrectomy | Creat (days) after nephrectomy |
| --- | --- | --- | --- | --- | --- |
| 1 | 75 | F | Renal Cancer | 82 (39) | 108 (1) |
| 2 | 61 | M | Renal Cancer | 66 (41) | 98 (1) |
| 3 | 75 | F | Renal Cancer | 65 (1) | 93 (1) |
| 4 | 49 | M | Renal Cancer | 59 (1) | 91 (1) |
| 5 | 73 | M | Renal Cancer | 72(2) | 89 (1) |
| 6 | 62 | F | Renal Cancer | 87 (on OR day, pre-OR) | 94 (1) |
| 7 | 79 | M | Renal Cancer | 74 (1) | 239 (1) |
| 8 | 20 | F | Renal Cancer | 80 (23) | 424 (1) |
| 9 | 44 | M | Renal Cancer | 92 (20) | 197 (1) |
| 10 | 56 | F | Renal Cancer | 63 (on OR day, pre-OR) | 91 (1) |
| 11 | 73 | F | Renal Cancer | 58 (1) | 54 (1) |
| 12 | 47 | F | Renal Cancer | 114 (15) | 99 (8) |
