# Supplementary material for: Kidney histopathology in lethal human sepsis
Source: Crit Care. 2018 Dec 27;22:359. doi: 10.1186/s13054-018-2287-3 (PMC6307291; doi:10.1186/s13054-018-2287-3)
Supplement: Supplementary file 2 — Supplementary methods and results. (PDF 78 kb) [file 13054_2018_2287_MOESM2_ESM.pdf]

Additional file 2

Kidney histopathology in lethal human sepsis: Heterogeneous damage and simultaneous repair

Adnan Aslan[1,2], Marius C van den Heuvel[3], Coen A Stegeman[4], Eliane R Popa[2], Annemarie M Leliveld[5], Grietje Molema[2], Jan G Zijlstra[1*], Jill Moser[1,2], Matijs van Meurs[1,2]

University of Groningen, University Medical Center Groningen

[1]Department of Critical Care

[2]Department of Pathology & Medical Biology, Medical Biology section

[3]Department of Pathology & Medical Biology, Pathology section

[4]Department of Nephrology

[5]Department of Urology

Hanzeplein 1

9700 RB Groningen, Netherlands

Correspondence: * Jan G. Zijlstra, MD, PhD, BSc, University Medical Center Groningen, University of Groningen, P.O. 30.001, 9700 RB Groningen, Netherlands.

E-mail: j.g.zijlstra@umcg.nl

Supplemental Table 3: Primary antibodies

Target antigen	target	clone	source
Neutrophil elastase	Neutrophils	68672	Abcam, Cambridge, UK
CD3	Pan-T-lymphocytes	2GV6	Ventana Medical Systems, Tuscon, TX
CD4	Helper T-lymphocytes	SP35	Ventana
CD8	Cytotoxic T-lymphocytes	C8/144B	DAKO Heverlee, BE
CD20	B-lymphocytes	L-26	Ventana
CD68	Pan-macrophages	PG-M1	DAKO
IRF5	Type I macrophages		Proteintech, Manchester, UK
CD163	Type II macrophages	NCC-CD163	Novacastra Reagents, Leica, Eindhoven, NL
a-SMA	Myofibroblasts/SMC	1A4	Ventana
Ki-67	proliferation	30-9	Ventana
Act. caspase 3	apoptosis	9661	Cell Signaling, Danvers, MA

Supplemental Table 4: Histopathological evaluation of glomeruli and tubulointerstitium

Structure	Evaluation
**Glomeruli**	
Total count (of all 27 patients)	926
Sclerotic (absolute count; percentage)	31 (3.35%)
Glomerulitis	None
Increase of mesangial matrix	All stage 0
**Tubulointerstitium**	
Tubulitis	None
Interstitial inflammation	2 (both patients stage 1)
Interstitial fibrosis	7 (all 7 patients stage 1)
Tubular atrophy	8 (all 8 patients stage 1)
**Vessels**	
Intima sclerosis	20 (13 patients stage 1, 7 patients stage 2)
Arteriolar hyaline	14 (12 patients stage 1, 2 patients stage 2)
Intima arteritis	None
Peritubular capillaritis	All patients stage 0

Supplemental Table 5 Presence of acute tubular necrosis

	Sepsis	Control
**Signs of ATN** (Yes/No)	24/3	0/12
**Morphology**		
Stage 1	4	
Stage 2	18	n.a.
Stage 3	2	
**Extensiveness**		
Stage 1	19	
Stage 2	2	n.a.
Stage 3	2	
Stage 4	1	

n.a: not applicable

Figure S1

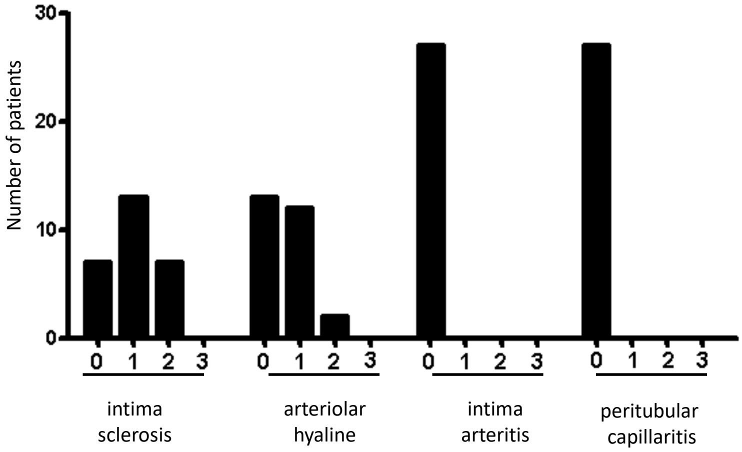

30
20
10
0
Number of patients
0 1 2 3
0 1 2 3
0 1 2 3
0 1 2 3
intima sclerosis
arteriolar hyaline
intima arteritis
peritubular capillaritis

Supplemental figure legend

Figure S1: Vascular abnormalities

Intima sclerosis (%vessel lumen narrowing)

0: none; 1: -25%; 2: 25-50%; 3: >50%

Arteriolar hyaline

0: none, 1: small-moderate, 2: moderate-severe in multiple arteries, 3: severe in most arteries

Intima arteritis

0: none; 1: -25% vessel lumen narrowing; 2: >25% vessel lumen narrowing; 3: transmural arteritis, fibrinoid changes and media necrosis

Peritubular capillaritis

0: <10% capillaries containing inflammatory cells; 1: >10% capillaries containing 3-4 luminal cells; 2: >10% capillaries containing 5-10 luminal cells; 3: >10% capillaries containing >10 luminal cells
